# The Treatment of Malignant Mammary Neoplasma

**Published:** 1897-12

**Authors:** Edward B. Jackson

**Affiliations:** Houston, Texas


TEXAS MEDICAL JOURNAL.

ESTABLISHED JULY, 1885.

PUBLISHED MONTHLY.—SUBSCRIPTION $2.00 A YEAR.

VOL. XIII. AUSTIN, DECEMBER, 1897. NO. 6.

### Original Contributions.

For the Texas Medical Journal.

### THE TREATMENT OF MALIGNANT MAMMARY NEOPLASMA.

BY EDWARD B. JACKSON, M. D., HOUSTON, TEXAS.

The appalling prevalence of scirrhous cancer, and sarcomatous growths of the breast demands the earnest attention of every general practitioner, since it is he who, in the majority of instances, first meets with the disease; and if, for any cause, there should be fatal delay in applying correct measures, a great and everlasting responsibility is incurred.

It is true that excision of the mamamry gland is classed among major operations, but we are going to try to prove that its technique, and the pathological conditions requiring its execution, are so easy of understanding that no physician, however remote or rural, should be without the necessary intelligence and means of accomplishing it.

It is almost needless to draw renewed attention to the very important surgical canon, namely—the positive necessity of early operation in all cases where there is suspicion of malignancy.

It would be well to remember, too, upon all occasions, that in the beginning of cancer there is only the sole symptom—pain. The other diagnostical characters, which are obtainable by inspection and palpation, are developed always later. Pain in the breast should, therefore, be invariably considered a sufficient symptom to put the physician upon constant watch.

Probably the most important point in connection with excision of the breast for malignant neoplasm, is the very rigid care necessary in the removal of not only the entire mass of active, spreading disease, but a sufficient amount of adjacent healthy structure to make sure that the line of necrotic invasion has been fully embodied in the amputated product.

It is believed to be always wise to remove the breast in its entirety, no matter how recent or restricted the lesion; and to this end it is expedient, in the process of enucleation, to keep the dissections well on the outside of the capsule of the gland, using the fingers freely in detaching the mass from the outlying structures.

The fact that the breasts are organs which are subject to involution changes, explains, in great measure, the frequency with which they are the seat of malignant disease; and it is doubtless because of the easy extension and reproduction of the lesion in such tissue that the operation of complete removal is justifiable. There has been also abundant evidence to show that the breasts are more liable to scirrhous cancer than any other portion of the body; and that, when attacked, the disease more rapidly invades their entire structure; and further, that they may be hopelessly invaded even when there are are no evidences of disease to the eye or touch. This, coupled with the fact that a portion of the gland, even if left behind, would be useless and unsightly, makes it quite clear that complete excision is the preferable procedure. There may be, of course, occasionally some special cases in which, when it is specially requested, and when it is within the bounds of reason, a certain amount of curtailment in operative measures may be practiced; though it has been the common experience of surgeons that, to leave a portion of the gland behind, for any cause, is usually fraught with early disastrous consequences, requiring eventually a repetition of surgical interference. In the performance of the operation of excision of the breast, an elliptical area of skin including the areola and nipple, must be removed, in order to facilitate completeness of gland extirpation, and to leave a linear eschar in a position to be as little acted upon as possible by muscles of the locality.

If the skin, however, should be widely infiltrated and extensively adherent, it must be more liberally included in the incision, without any direct reference to healing by first intention, or the production of a well located and pleasing scar; since if the smallest infected fragment remains in the wound, the malignant disease will proceed uninterruptedly upon its course of devastation. It is also wise, we believe, upon all occasions, particularly when the entire gland is infected, to remove the underlying pectoral fascia; and if there is any evidence of subjacent muscular infection the pectoralis major should be likewise liberally excised. The uppermost idea in the operator's mind should ever be in the direction of clearing away every whit of the diseased tissue, even at the expense, where there is reasonable doubt, of occasionally sacrificing healthy structure.

The chain of lympathics, which passes through the band of fatty tissue between the breast and arm-pit, together with the axillary glands, and all of the surrounding bands of fat, should be detached from the pectoral and serratus magnus muscles, from the subscapular fascia, and from the axillary nerves and vessels with the fingers, aided occasionally by a raspatory, taking care, of course, not to wound the intercosto-humeral nerve, the acillary vein, and the cutaneous branches of the intercostal nerves.

It is granted that such a free removal of tissues would not be justifiable if we could know in advance that it were particularly free of disease; but since it is manifestly impossible to make sure of its healthful condition, until viewed microscopicaly; and in so much as such structures so easily partake of the morbid process, and are frequently fatally infected when they are to the unaided eye apparently healthy, it is to be recommended as a justifiable procedure, particularly applicable to all cases of scirrhous cancer.

We are aware, however, of contrary opinions entertained by some well known surgeons for whose teachings, otherwise, we have the greatest respect.

Certainly the axillary glands, even when healthy, cannot be of any very great service when left behind, since the chain of lymphatics which supplies them is broken by the mammary dismemberment. We have felt them carefully upon several occasions when they seemed perfectly healthy, and have found, deeper down, in the pillows of fat, after taking them away, one or more hard knots of growing disease, which, had they been left there, would soon have been considered so-called "recurring cancer." We believe, too, that there is no excuse for not removing diseased adjacent glands of the neck, unless they be so extensively attached to large trunk vessels as that they can not by any means be exsected.

The surgeon in the consideration of shock, sepsis and hemorrhage should not allow himself to make the serious and inexcusable mistake of taking away too little tissue, or of uselessly interrupting his incision; since if a chain of glands be infected the chances are that the surrounding structures are not entirely free from the morbid process; and such parts should therefore be included in the incision in order to bring them into full view for examination.

It is seen, therefore, that an incision when begun should be followed up as long as there is infection in its wake.

The union by first intention of an incision extending from the breast to the axilla occurs as readily, in all cases where co-aptation is easily and symmetrically obtained, as though separate shorter incisions were made. Besides it would be, of course, impossible to reflect from the chest-wall the considerable adipose cushion connecting the breast with the axilla unless the incision was continuous—and the great majority of operators are fully agreed that this band of fat should come away before the operation can be considered complete. The risk of sepsis, shock, and so forth, is probably not at all increased; and there is, as every one admits, some less danger of local recurrence.

While it is of the greatest importance to check all hemorrhage and oozing, before attempting to close the wound, it should be accomplished if possible without the use of ligatures, since then a slight danger of *immediate* irritation is avoided. Usually nothing further than torsion and hot water is required to completely stanch every bleeding point. The hot water should preferably contain one part of corrossive sublimate to two thousand; and if, then, it be freely douched through the wound there may not, as sometimes otherwise happens, be an excessive and objectionable amount of serous exudation.

In view of the great difficulty with which perfectly aseptic sutures are obtained, and particularly maintained, the author is in the habit of using silver and silk-worm-gut; placing them, whenever the flaps meet easily, invariably and superficially. And in great many instances when there is apparently a considerable degree of tension, it can often be relieved by a system of bandaging, and specially applied adhesive strips.

In this way, by foregoing deep sutures or pins, the danger of infecting probably the entire wound, setting up extensive suppuration and sloughing, is avoided. It is easy to see how the dermal covering, already somewhat attenuated by pressure of the tumor, may, when rendered too taut, and constricted by deep sutures or pins, break down and slough away,—whereas had it been brought together only in such places as would meet easily by superficial sutures, the intervening gaps would have rapidly healed in the wake of the interspaces of primary union.

If the operator can make sure he has removed all of the disease, and will take special pains to see that the wound is rendered perfectly aseptic, he need not insert a drainage tube; for under such circumstances it is not only entirely useless, but cumbersome and objectionable, and not without a degree of danger; since in order to accomplish its removal on the second day, or later, the wound will have to be in a measure opened, and thus subjected to the peril of infection from the air.

There are some occasional cases however, in which for obvious reasons the drainage tube will be a most wise precautionary step.

After the wound has been closed it is good practice to dust its crevices, along the line of apposition and round about the suture punctures, with an antiseptic, nonirritating powder, and for this purpose we have found all of the required virtues in sennine.

The wound should now be covered with several layers of sublimate gauze, followed by a thick pad of carbolized absorbent cotton, to be kept in place by a carefully adjusted roller bandage.

Over all of this dressing should be carried a wide domestic sling, binding the arm, at right angle, firmly to the body. Underneath the elbow of the affected side a pillow should be placed for support, until the patient is able to be propped up in bed, which is, as a rule, not later than the third day. After the fifth day, if provisions for a comfortable chair or cot are made, the bed may, during the day time, be abandoned altogether. The wound should be examined on the seventh day; and very often, at this stage, the union will be found so complete that all of the sutures may be removed; but occasionally, when healing, foı any cause, has proceeded more slowly, it may be the part of wisdom to remove only every other stitch, placing also, a narrow strip of court plaster in its stead, to guard the line of tender adhesion, until, in one or two days more, as the case may be, all of the remaining sutures are thus removed.

Sometimes we have had the patient complain of pain on the fifth day, and have found on opening the dressing that one or more stitches were cutting the tissue. In such instances we have merely clipped one side of the constricted sutures, under the knot, and allowed them to remain until the next dressing—say two days later.

The new and friable cicatrix can bear but little strain, and should, therefore, be protected with adhesive strips. It is believed that the necessity of bearing this point well in mind is worthy of special emphasis, since upon two occasions after the last sutures were removed on the eighth day, the writer, from neglect of it, had the chargin of witnessing wide gaps and the discomfort of having to treat them through the tedious process of healing by second intention, requiring weeks of constant care.

Some of the authors who claim that the healing of localities, recently infected with malignant disease, by extensive suppuration is rather more favorable than otherwise, may say that such accidents are not to be greatly regretted; but we know that the super-abundance of opinion declares that the suppurative and granulative processes do not in the least degree tend to render tissues less prone to the recurrent growth of cancer.

A safe rule therefore is, (1) to remove every vestige of disease, no matter how extensive; and (2), get union invariably at the earliest possible moment. If from any cause it is impossible to obtain primary union, the most reliable means of obtaining rapid secondary healing should be instantly applied; and of such measures we are convinced there are none, save Thierschs' method of skin graft, so pregnant with energy and dispatch as the systematic application of an antiseptic powder, after washing twice a day with 1 to 2000 permanganate potassa solution. When the patient is well, and ready to be dismissed, the great mistake of promising too much should never be made. The patient should be given an intelligent understanding of the outlook, and should be requested to keep in constant touch with competent surgical skill, so that upon the supervention of the veriest suspicious lump upon any part of the body, it may, if necessary, be excised while yet minute. The surgeon can not, unfortunately, promise that the disease will never recur, and he should therefore seek, as a matter of self protection, to throw such light upon the possible conditions which may later be developed, as that, under any set of circumstances, his patient will feel free in confidently applying to him through even many direful outbreaks, with the hope of receiving kind, correct and reasonable treatment. These patients need to be taken largely into our confidence; they should be always encouraged to listen to reason, they require earnest and constant interest, and renewed daily consideration, otherwise, when they see the surgeon wearing a hopeless countenance; when they realize he no longer cares to be troubled with their case, they are sure to fall in the hands of some unscrupulous mountebank. We are in duty bound, therefore, to treat, patiently, all hopeless cases until the very end; with no less earnestness too, than were they of such character as that we might advantageously use the knife and accomplish brilliant results. We should not be dominated by the hope of success alone. We should make ourselves what we ought to be, and everything else will then be well. One further word in reference to inoperable cases; we have tried parenchymatous injections of blue pyoktanin according to Meyer and Von Mosetig in doses of one and one-half cubic centimeters, two per cent solution, and we have tried inoculations of virulent cultures of streptococus erysipelatis according to Coley and Fehleisen which were in due time followed by chill, reaction, and all the concomitant symptoms of violent erysipelas; and we have after all only met with defeat.

According to directions above given in this paper the surgeon must *continue to treat* every case; and in order to maintain the confidence of the patient he must *be doing something;* and we therefore strongly insist when all else has failed that the arsenious paste,—exact proportions and directions found in Wyeth's Text Book—be applied. It contains cocaine in quantity to render its action painless. The reader is referred to the text-book for explicit instructions, since here we have not the time or space to give it.

I recommend the 1 in 2000 solution of potassa permanganatis as the best and quickest means of dispelling the sickening odor from the room and disinfecting the putridity of the malignant lesion. This solution should be sprayed in the room, exposed in saucers, and applied upon a great pad of cotton wool thoroughly saturated in direct contact with the cancerous ulcer. It deodorizes and disinfects with incomparable energy. And there is great comfort in knowing that toxic symptoms will not accrue from its use. There is no remedy which will so soon thwart the stench of the sore and bring back purity to viciated air.

